# Comparative Study of Physicochemical and Structural Characteristics of Meat Analogues Produced From Soy and Wheat Proteins

**DOI:** 10.1002/fsn3.70780

**Published:** 2025-08-12

**Authors:** Flora farrokhi, Mohammad Hossein Azizi

**Affiliations:** ^1^ Department of Food Science and Technology Tarbiat Modares University Tehran Iran

**Keywords:** extrusion, fibrous structure, plant‐based meat, soy protein isolate, wheat gluten

## Abstract

Plant‐based meat analogues are gaining attention due to rising environmental concerns and demand for sustainable dietary options. However, achieving desirable texture and structure remains a challenge. This study investigated how varying proportions of soy protein isolate (SPI) and wheat gluten (WG) affect the physicochemical, structural, and sensory properties of high‐moisture extruded meat analogues. SPI and WG were blended in different ratios (0%, 15%, 25%, 40% WG) and processed using a twin‐screw extruder under controlled conditions. Additional ingredients included 7% vegetable oil, 5% pumpkin powder, 3.7% wheat starch, and 0.3% salt to enhance structure, color, and palatability. Physicochemical, textural, microstructural (SEM), and sensory analyses were conducted. Formulations containing 40% WG exhibited superior fibrous texture, hardness, chewiness, and flexibility. SEM analysis showed increasing WG content enhanced fiber density and alignment; however, the ultrastructure (defined here as sub‐micrometer fibrous morphology) remained consistent in type. Protein solubility results suggested that hydrogen bonds, disulfide bridges, and hydrophobic interactions stabilized the network. Combining SPI and WG under high‐moisture extrusion enhances the fibrous characteristics of meat analogues. Optimizing the WG content leads to improved texture and structural integrity, offering a viable strategy for developing consumer‐acceptable, sustainable meat substitutes.

## Graphical abstract

High‐moisture extrusion of soy protein isolate (SPI) and wheat gluten (WG) blends (0%–40% WG) revealed that 25%–40% WG optimally enhances fibrous texture and structural integrity. SEM imaging confirmed denser fiber alignment with higher WG, while sensory analysis validated improved meat‐like texture. This work provides a scalable strategy for sustainable plant‐based meat production.

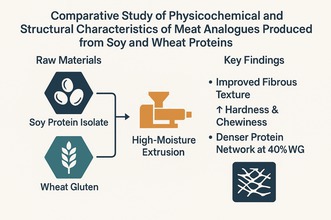

## 1 Introduction

The growing interest in plant‐based meat analogues is fueled by environmental, ethical, and health‐related concerns (Zhang et al. 2023). Despite substantial progress, replicating the complex texture and mouthfeel of conventional meat remains a significant challenge. Soy protein isolate (SPI) and wheat gluten (WG) are widely utilized in high‐moisture extrusion processes due to their capacity to form fibrous structures resembling animal muscle tissue.

While advances in plant protein processing are promising, achieving parity with animal‐derived meats in terms of flavor depth, tenderness, and nutritional value remains elusive. Research by Sha and Xiong (2022) underscores the pivotal role of extrusion in enhancing meat analogue structure by examining how different proteins behave under processing conditions.

In addition to technological innovation, consumer acceptance plays a critical role. Michel et al. (2021) emphasize that sensory properties, health perceptions, environmental benefits, and cultural preferences all shape consumer decision‐making. Aligning product profiles with these expectations is essential to facilitate mainstream adoption. As global demand for sustainable food alternatives rises, plant‐based meat analogues are positioned as a potential solution to reduce the ecological footprint of traditional meat production.

Studies by Tziva et al. (2019) and Curtain and Grafenauer (2019) highlight the relevance of meat substitutes in emerging dietary practices, particularly among flexitarians who aim to reduce but not eliminate meat consumption. Nevertheless, current plant‐based offerings represent only a small share of the meat market—primarily due to unresolved challenges in recreating authentic taste and texture (Grabowska et al. 2014; Krintiras et al. 2015).

Diverse plant proteins—including those from soy, wheat, pea, rice, and corn—exhibit unique functional and sensory characteristics that shape the development of meat analogues (Muthukumarappan & Karunanithy, 2012). Innovating through novel protein combinations and refining extrusion conditions offers considerable potential to improve product quality. Moreover, formulations catering to consumers with dietary restrictions (e.g., gluten‐free or soy‐free) can broaden market accessibility (Malav et al. 2015; Ild et al., 2014).

Protein modification via hydrothermal treatment during extrusion is instrumental in texture development. As proteins denature, realign, and form new bonds, structural integrity is enhanced—particularly in the cooling die section, where fibrous alignment is reinforced and steam‐driven expansion is minimized (Day and Swanson 2013; Golbitz et al. 2006). High‐moisture extrusion facilitates these transformations, with SPI promoting fiber formation and WG contributing to elasticity and matrix reinforcement (Chen et al. 2010; Fang et al. 2014).

In this study, a formulation consisting of 7% vegetable oil, 5% pumpkin powder, 3.7% wheat starch, and 0.3% salt was employed to enhance the texture, color, flavor, and mouthfeel of extruded analogues. Pumpkin powder was specifically selected for its natural pigmentation, mild taste, and antioxidant potential—supporting both visual and sensory appeal.

Although prior research has underscored the significance of SPI and WG in extrusion, limited studies have explored how incremental changes in WG concentration affect macro‐ and microstructural development. Addressing this gap, the present study aims to investigate the impact of varying SPI–WG ratios on the textural, physicochemical, microstructural, and sensory attributes of extruded plant‐based meat analogues. The findings are intended to inform optimal formulation strategies for producing sustainable, high‐quality alternatives to conventional meat.

## 2 Materials and Methods

### 2.1 Materials

Soy protein isolate (ALPHA 11 IP, Solae) containing 70.2% protein, 4.8% moisture, 4.4% ash, 18.8% carbohydrate, and 1.8% fat was sourced from Tari International NZ Ltd. (Auckland, New Zealand). Wheat gluten (FLOURG25, 75% protein, 10% moisture, 1.5% ash, 12.5% carbohydrate, and 1% fat) and wheat starch (FLOURCW25, 0.4% protein, 12.1% moisture, 0.5% ash, and 87% carbohydrate) were obtained from Davis Trading (Palmerston North, New Zealand). All chemicals and reagents used in the study were of analytical grade.

### 2.2 Methods

#### 2.2.1 Preparing the Samples by High‐Moisture Extrusion Process

High‐Moisture Extrusion Process Following methods outlined by Chen et al. (2010) and Fang et al. (2014), samples were processed using a twin‐screw extruder (Clextral BC‐21, Firminy Cedex, France) featuring pilot‐scale, co‐rotating, and intermeshing capabilities. The formulations for meat analogues were developed with varying soy protein isolate (SPI) to wheat gluten (WG) ratios, comprising 0% WG (84:0), 15% WG (69:15), 25% WG (59:25), and 40% WG (44:40). Each mixture included 7% vegetable oil, 5% pumpkin powder, 3.7% wheat starch, and 0.3% salt. The choice of additives was based on functional and sensory roles: vegetable oil for fat content and mouthfeel, pumpkin powder for natural color and antioxidant properties, wheat starch as a binder and moisture retention agent, and salt to enhance flavor.

Extrusion process variables were controlled with: Length‐to‐diameter ratio of screw (𝐿𝑠/𝐷𝑠) = 28:1, Barrel diameter (𝐷𝑏) = 26 mm, Screw diameter (𝐷𝑠) = 24 mm, Total screw length (𝐿𝑠) = 690 mm, Cooling die dimensions = 10 mm/355 mm. The extruder screw profile comprised various forward screws and one reverse screw, optimized to ensure effective protein alignment and structure formation. The feeding zone (T1) and barrel were connected, with six steam‐heated zones (T2 to T7) maintained under controlled temperatures ranging from 20°C to 170°C, cooled with ambient water. Ingredients were introduced via a gravimetric feeder (Coperion K‐Tron, Switzerland) at 2.8 kg/h, while water injection into the system was set at 3.6 kg/h, maintaining an overall moisture content of 60% (wet basis) in the final extrudates. The screw rotation speed was 400 rpm, ensuring uniformity during processing.

#### 2.2.2 Physicochemical Analysis

Protein content was measured using the Kjeldahl method (Foss Tecator Inc., Sweden), multiplying nitrogen content by 6.25 for SPI and meat, and by 5.7 for WG. Moisture content was determined following Nielsen (2017), with modifications, using heated aluminum pans (108°C, 1 h) and a 24‐h drying process. pH levels were assessed using a Mettler‐Toledo SG23 pH meter, calibrated with standard buffer solutions (pH 4 & 7) per Liu and Hsieh (2007). Additional physicochemical analyses included testing for aflatoxins, pesticide residues, and heavy metals (Chen et al. 2010; Fang et al. 2014).

#### 2.2.3 Textural Properties—Cutting Force, Hardness & Chewiness

Texture measurements were conducted using a TA.XT Plus texture analyzer (Stable Micro Systems, UK), applying a knife blade probe to diced samples (15 × 15 × 8 mm). The degree of texturization (DT), indicating fibrous structure formation, was quantified via longitudinal (𝐹𝐿) and crosswise (𝐹𝑉) strength measurements. Hardness and chewiness assessments used a two‐bite compression test, following Fang et al. (2014).

#### 2.2.4 Microscopy—Scanning Electron Microscopy (SEM) Analysis

Samples (10 × 10 × 10 mm) underwent fixation using Modified Karnovsky's fixative (3% glutaraldehyde, 2% formaldehyde, 0.1 M phosphate buffer, pH 7.2). Dehydration involved a graded ethanol series (25%, 50%, 75%, 95%, 100%), followed by critical‐point drying with liquid CO_2_. Samples were sputter‐coated with 100 nm gold (Baltec SCD 050) and examined via Quanta 200 Environmental SEM (FEI Co, USA) at 250× magnification, using 20 kV accelerating voltage (Fang et al. 2014).

#### 2.2.5 Microbial Investigation

Meat analogue samples (10 g) were homogenized in peptone water, prepared in 10‐fold dilutions, and plated on agar media: Aerobic colony count—PCA plates incubated at 30°C for 72 h (ISO 4833‐2:2013); Mold detection—DRBC agar incubated at 25°C for 5 days (ISO 21527‐1/2:2008); Enterobacteriaceae enumeration—VRBG agar incubated at 37°C for 24 h (ISO 21528‐2:2007).

#### 2.2.6 Sensory Evaluation

Ten trained panelists participated in a single‐blind sensory evaluation to assess fibrous structure, hardness, and flexibility. Samples (25 × 15 × 8 mm) were evaluated at ambient temperature, and panelists were provided water to cleanse the palate between samples. Evaluation criteria included: (1) Fibrous structure: visual tearing test (1 = no fibers, 9 = highly fibrous) ‐ Hardness: manual tearing test (1 = soft, 5 = firm, 9 = hard); (2) Flexibility: twisting test (1 = tender, 5 = flexible, 9 = tough).

Results were cross‐referenced with instrumental texture analysis to validate consistency and minimize subjectivity.

#### 2.2.7 Data Analysis

Experiments were conducted in triplicate, with results presented as means ± standard deviations. One‐way ANOVA (*p* ≤ 0.05) was performed using Minitab 16.2.1 (Minitab Inc., USA). Figures were generated via Origin Software 8.5 (Origin Lab Corp., MA, USA).

## 3 Results and Discussion

### 3.1 Physicochemical Properties of Individual Meat Analogues

**FIGURE 1:**
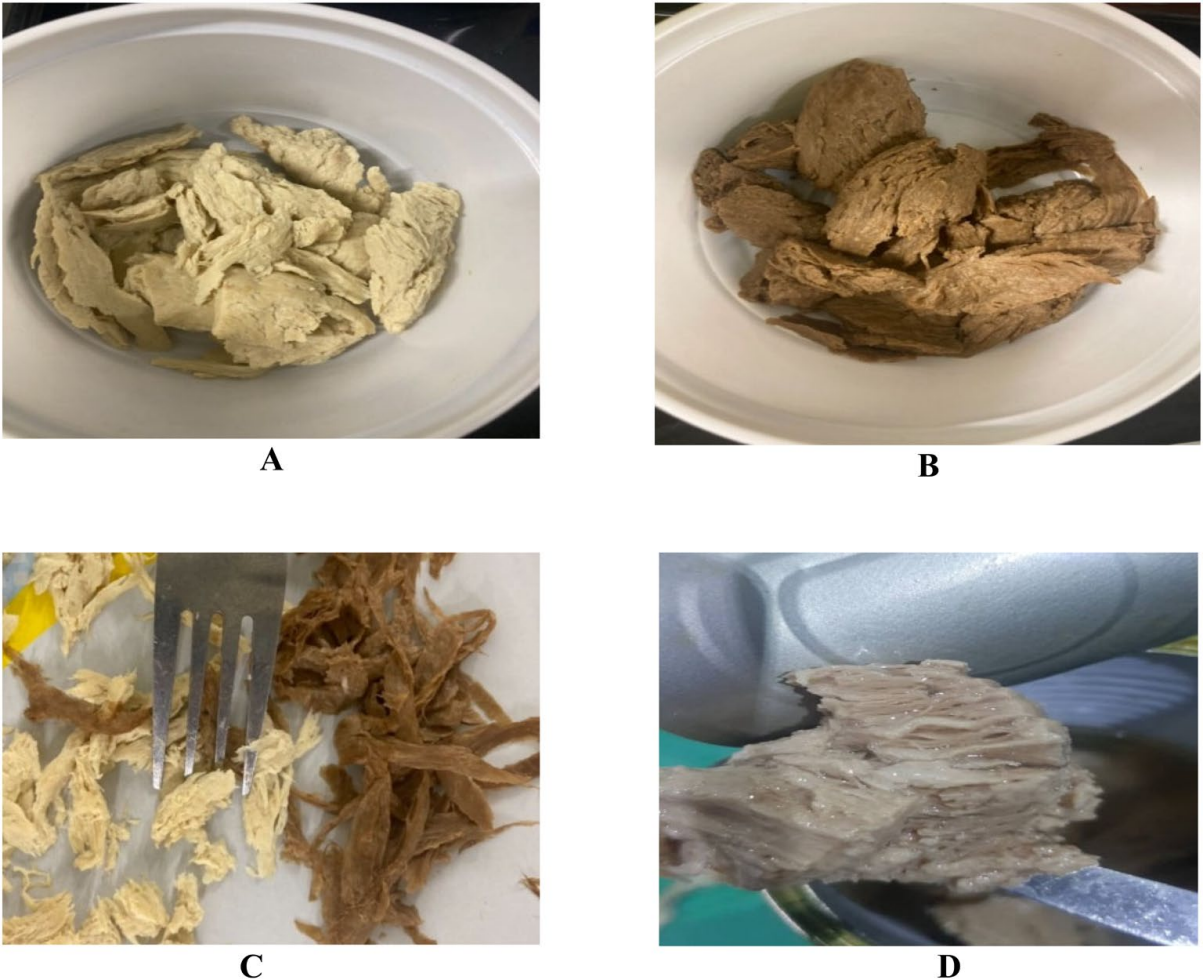
The visual images of different individual extruded meat analogues with different soy protein isolate and wheat gluten ratios: (A) 84:0 (0% WG), (B) 69:15 (15% WG), (C) 59:25 (25% WG), and (D) 44:40 (40% WG). Coloring agents were applied for visual enhancement; actual colorimetric analysis was conducted on uncolored samples.

The visual characteristics of extruded meat analogues, formulated with different soy protein isolate (SPI) to wheat gluten (WG) ratios, are illustrated in Figure 1. It is important to note that the colors depicted in these images were adjusted using natural coloring agents. However, the color analysis of the samples was conducted on their neutral intrinsic color before any modifications. The physicochemical and microbial analyses of individual meat analogues are presented in Tables 1, 2, 3: Table 1 outlines protein content, moisture content, pH, and color properties. Table 2 provides a microbial profile, summarizing the average microbial loads across samples. Table 3 details aflatoxin levels, pesticide residues, and heavy metal concentrations. For comparative evaluation, Table 4 presents the protein content (% w/w of wet material), moisture levels, pH values, and L* values across various SPI‐WG formulations.

**TABLE 1:** Total analysis of average physicochemical properties of individual meat analogue samples.

Row	Properties		Results	Limited range
1	pH	SPI	6.96	6.8–7.8
		WG	6.73	
2	Protein (dry basis) (% w/w)	SPI	75.32	50–64.9
		WG	70.11	
3	Fat (dry basis) (% w/w)	SPI	2.67	2.50
		WG	3.04	
4	Total Ash (% w/w)	SPI	4.01	Less than 8
		WG	3.61	
5	Moisture (% w/w)	SPI	8.99	Less than 9
		WG	8.62	
6	Fiber (% w/w)	SPI	1.47	Less than 6
		WG	1.36	

**TABLE 2:** Total analysis of average microbial properties of soy and wheat meat analogue samples.

Row	Properties	Results	Limited range
1	Mold (cfu/g)	Less than 10	Less than 100
2	Total Count (cfu/g)	300	Less than 10,000
3	*Coliform* (cfu/g)	Less than 10	Less than 10
4	*E‐Coli* (cfu/g)	Negative	Negative
5	*Salmonella* (cfu/25 g)	Negative	Negative

**TABLE 3:** Total analysis of average chemical properties of soy and wheat meat analogue samples.

Row	Properties		Results	Limited range	LOQ
1	Aflatoxins (ng/g): B & G KHA—S001 Based on INSO6872 ver1	Aflatoxin B1	ND (Not detected)	Less than 25	1
		Aflatoxin B2	ND (Not detected)		0.2
		Aflatoxin G1	ND (Not detected)		1
		Aflatoxin G2	ND (Not detected)		0.2
		Total Aflatoxins B & G	ND (Not detected)		0.2
2	Pesticides Residue (mg/kg): GC/MS KHA—S041 Based on INSO17026		ND (Not detected)	—	—
3	Heavy Metals (mg/kg)	Lead	0.117	Less than 0.1	0.05
		Cadmium	0.040		0.02

**TABLE 4:** Protein content (% w/w of wet material), moisture content, pH level, and L* value of extruded meat analogues at different soy protein to wheat gluten ratios.

Sample	Protein content (%)	Moisture content (%)	pH	Color (L* value)
0% WG	26.42 ± 0.57^a^	53.11 ± 0.18^a^	7.29 ± 0.47^b^	56.14 ± 1.32^a^
15% WG	26.97 ± 0.41^a^	53.49 ± 0.14^a^	7.18 ± 0.12^b^	62.27 ± 1.15^bc^
25% WG	27.15 ± 0.70^b^	55.27 ± 0.10^b^	7.01 ± 0.09^ab^	62.86 ± 1.06^c^
40% WG	28.46 ± 0.16^c^	56.19 ± 0.30^c^	6.74 ± 0.36^a^	61.67 ± 0.92^b^

*Note:* Data are presented as the mean and standard deviation of three replicates. Values with different lowercase letters were significantly different (*p* ≤ 0.05).

Protein, Moisture, and pH Levels The protein content across all meat analogues ranged between 26.42% and 28.46%. Moisture content varied from 53.11% to 56.19%, slightly below the targeted 60% moisture level reported in the literature (Warner, 2017). The observed moisture loss was attributed to protein denaturation at high temperatures, which induces transverse and longitudinal shrinkage during extrusion (Warner, 2017).

The pH values of the analogues were between 6.74 and 7.29. While statistically significant differences were observed among samples, they were considered minor in practical terms (Fletcher et al. 2000).

Color Analysis Regarding color parameters, increasing the WG concentration corresponded to an increase in L* values (lightness), ranging from 56.14 to 61.67. Analogues formulated with 40% WG exhibited the highest brightness, indicating an effect of WG on visual appearance. The color brightness (L*) values increased with WG content. This is likely due to WG's lighter color compared to SPI and its lower propensity for Maillard browning. The enhanced brightness also reflects reduced protein aggregation, which may allow more uniform light scattering.

### 3.2 Effects of SPI to WG Ratio on Textural Properties

The degree of texturization (DT) for various SPI‐WG ratios is presented in Table 5. DT (𝐹𝐿/𝐹𝑉) quantifies fibrous structure formation, with values greater than 1 indicating enhanced fiber alignment (Chen et al. 2010). Influence of SPI‐WG Ratio on DT As WG levels increased, DT values rose proportionally. The highest DT values were observed in samples containing 40% WG, reinforcing the role of WG in fibrous texture development. Compared to previously reported values (Fang et al. 2014; Chen et al. 2010), SPI‐WG formulations exhibited greater DT improvements than SPI‐only analogues. Mechanical Energy and Textural Properties Extrusion‐specific mechanical energy (SME) values decreased as WG content increased: 0% WG: 824 kJ/kg, 15% WG: 751 kJ/kg, 25% WG: 611 kJ/kg, 40% WG: 503 kJ/kg.

**TABLE 5:** Textural (instrumental) properties of meat analogues at different soy protein to wheat gluten ratio.

Sample	Degree of texturization (DT)	Hardness (N)	Chewiness (N)
0% WG	1.44 ± 0.11^a^	50.11 ± 1.14^a^	32.12 ± 1.32^a^
15% WG	1.50 ± 0.27^ab^	51.25 ± 5.22^a^	35.25 ± 3.17^b^
25% WG	1.68 ± 0.63^b^	59.76 ± 3.47^b^	40.50 ± 2.63^c^
40% WG	1.92 ± 0.29^c^	76.14 ± 5.26^c^	47.19 ± 4.08^d^

*Note:* Data are presented as the mean and standard deviation. Values bearing different lowercase letters were significantly different (*p* ≤ 0.05).

A double‐compression test (Textural Profile Analysis, TPA) was performed (Bourne 2002), evaluating: Hardness (maximum force during compression), Chewiness (𝐻𝑎𝑟𝑑𝑛𝑒𝑠𝑠 × 𝐶𝑜ℎ𝑒𝑠𝑖𝑣𝑒𝑛𝑒𝑠𝑠 × 𝑆𝑝𝑟𝑖𝑛𝑔𝑖𝑛𝑒𝑠𝑠) (Bourne 2002; TTC, 2016).

SPI‐dominant formulations resulted in lower chewiness and hardness, whereas increased WG incorporation enhanced fibrous structure formation, reducing SME and modifying textural integrity.

### 3.3 Sensory Analysis

**FIGURE 2:**
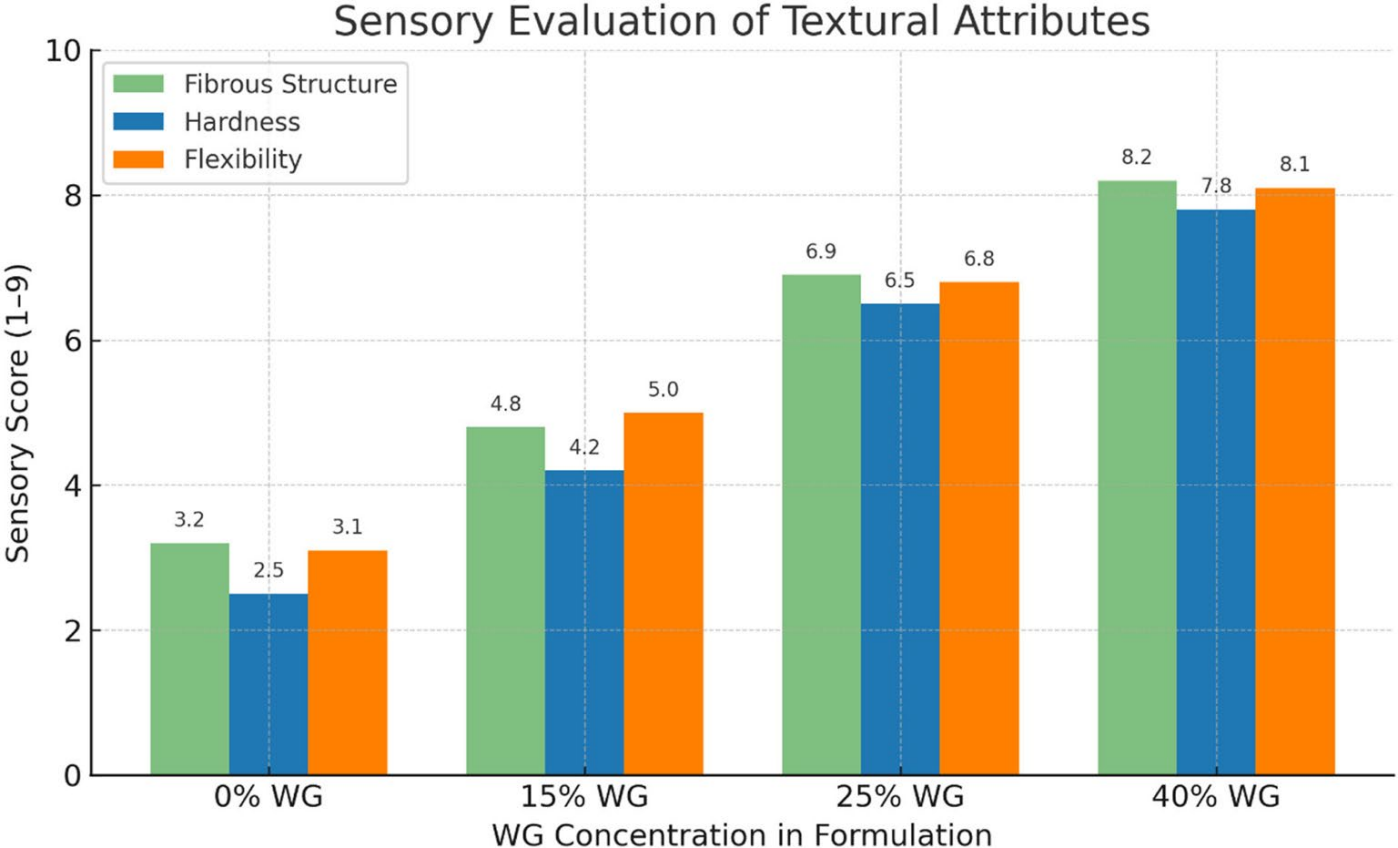
Sensory evaluation of textural attributes (fibrous structure, hardness, and flexibility) of extruded meat analogues formulated with varying wheat gluten (WG) concentrations. Values represent the mean sensory scores from 10 trained panelists (scale: 1 = least, 9 = most).

To further examine textural differences, a sensory evaluation was conducted (Figure 2); assessing: Fibrous structure (visual tearing test), Hardness (manual tearing test), Flexibility (twisting test).

Panelists noted an increase in fibrous structure scores with higher WG incorporation. Significant differences were observed across formulations (0%–40% WG), supporting WG's impact on fiber formation. In contrast, a prior study comparing grilled chicken breast showed lower fibrous structure scores, suggesting consumer associations of “fibrous” with “tough” textures (Szczesniak, 2002). Increased sensory hardness and flexibility scores correlated with instrumental TPA results, confirming that SPI‐WG ratios influence textural differentiation.

### 3.4 Effects of SPI to WG Ratio on Microstructural Properties

**FIGURE 3:**
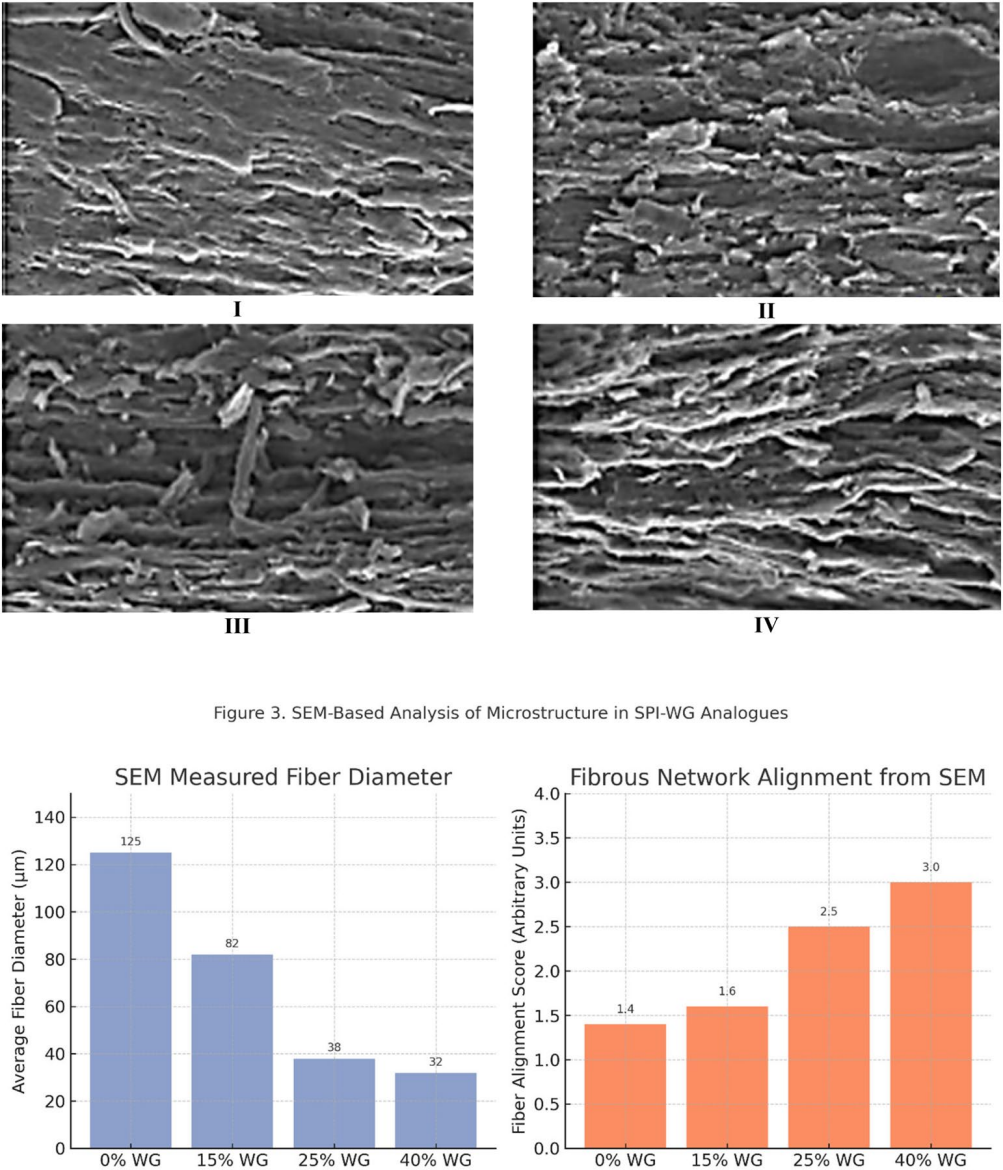
SEM micrographs (200 *μ*m) of extruded meat analogues with different soy protein isolate to wheat gluten ratio: (I) 0% WG, (II) 15% WG, (III) 25% WG, (IV) 40% WG, respectively, and SEM‐derived metrics of microstructural properties in extruded meat analogues with varying wheat gluten (WG) content. (Left) Average fiber diameter decreases with higher WG, indicating denser fibrillation. (Right) Fiber alignment score increases, reflecting enhanced structural organization. Data inferred from SEM image observations at 250× magnification.

SEM micrographs of meat analogue samples are presented in Figure 3. The observed microstructures varied across formulations: 0% WG: layered structures (100–150 μm), 15% WG: segmented layers (50–115 μm), 25% WG: fibrous microstructures (20–55 μm), 40% WG: dense fibrous networks (20–55 μm).

Comparison with Muscle Fibers SPI‐WG analogues containing 25% and 40% WG exhibited highly fibrous microstructures, akin to muscle fibers observed in SEM images of cooked chicken breast (Takei et al., 2016). Prior studies on 22.6% WG formulations (Krintiras et al. 2015) confirm that smaller fibers correspond to WG, reinforcing anisotropic structures in extruded analogues. Although the introduction noted that WG concentration had negligible effects on ultrastructure, SEM analysis revealed increased density and alignment of fibrous structures with higher WG levels. This is not a contradiction, as the type of ultrastructure remained similar, but its organization and compactness improved, particularly at 25% and 40% WG.

## 4 Conclusion

This study highlights the crucial role of wheat gluten (WG) in shaping the fibrous structure of meat analogues, demonstrating that higher WG incorporation enhances structural integrity. As WG levels increased relative to soy protein isolate (SPI), fibrous structure formation became more pronounced, with 40% WG formulations exhibiting the highest DT values, fibrous structure, hardness, and chewiness. From a structural perspective, meat analogues containing 25% and 40% WG proved significantly more acceptable compared to lower WG formulations, reinforcing the impact of protein interactions in determining textural quality.

Hydrogen bonds were identified as the primary stabilizing force, while disulfide bonds played a pivotal role in fibrous network formation. The enhanced brightness (L*) and reduced SME values also indicate energy‐efficient structural development.

These findings underscore the importance of optimizing WG‐SPI ratios in extrusion‐based meat analogue production; presenting valuable insights for developing plant‐based alternatives with enhanced structural and sensory characteristics.

Further studies should explore flavor optimization, long‐term shelf stability, and consumer acceptance under real‐world conditions.

## Conflicts of Interest

The authors declare no conflicts of interest.

## Data Availability

The authors have nothing to report.
